# Does the mental health system provide effective coverage to people with schizophrenic disorder? A self-controlled case series study in Italy

**DOI:** 10.1007/s00127-021-02114-9

**Published:** 2021-06-16

**Authors:** Giovanni Corrao, Angelo Barbato, Barbara D’Avanzo, Teresa Di Fiandra, Lucia Ferrara, Andrea Gaddini, Matteo Monzio Compagnoni, Alessio Saponaro, Salvatore Scondotto, Valeria D. Tozzi, Flavia Carle, Antonio Lora, the “QUADIM project”, “Monitoring, assessing care pathways (M. A. P.)” working groups of the Italian Ministry of Health, Teresa Di Fiandra, Natalia Magliocchetti, Antonio Lora, Miriam Barri, Alessio Saponaro, Andrea Gaddini, Valentina Mattia, Salvatore Scondotto, Walter Pollina Addario, Marco Berardi, Monica Di Giorgi, Giovanni Corrao, Matteo Monzio Compagnoni, Angelo Barbato, Barbara D’Avanzo, Igor Monti, Valeria Tozzi, Lucia Ferrara, Flavia Carle, Andrea Bucci, Chiara Casoli, Marianxhela Dajko, Donata Bellentani, Simona Carbone, Carla Ceccolini, Angela De Feo, Cristina Giordani, Lucia Lispi, Rosanna Mariniello, Federica Medici, Paola Pisanti, Modesta Visca, Rinaldo Zanini, Anna Cantarutti, Giovanni Corrao, Pietro Pugni, Federico Rea, Marina Davoli, Mirko Di Martino, Patrizia Vittori, Giuliana Vuillermin, Alfonso Bernardo, Anna Fusciante, Laura Belotti, Rossana De Palma, Enza Di Felice, Andrea Di Lenarda, Marisa Prezza, Danilo Fusco, Adele Lallo, Chiara Marinacci, Roberto Blaco, Olivia Leoni, Antonio Lora, Liana Spazzafumo, Simone Pizzi, Maria Simiele, Giuseppe Massaro, Ettore Attolini, Vito Lepore, Vito Petrarolo, Salvatore Scondotto, Giovanni De Luca, Paolo Francesconi, Carla Rizzuto, Francesco Avossa, Silvia Vigna, Letizia Dondi, Nello Martini, Antonella Pedrini, Carlo Piccinni, Mimma Cosentino, Maria Grazia Marvulli, Aldo Maggioni

**Affiliations:** 1 National Centre for Healthcare Research and Pharmacoepidemiology, University of Milano-Bicocca, Milan, Italy; 2 Unit of Biostatistics, Epidemiology and Public Health, Department of Statistics and Quantitative Methods, University of Milano-Bicocca, Street Bicocca degli Arcimboldi, 8, Building U7, 20126 Milan, Italy; 3 Unit for Quality of Care and Rights Promotion in Mental Health, Istituto di Ricerche Farmacologiche Mario Negri IRCCS, Milano, Italy; 4 General Directorate for Health Prevention, Ministry of Health, Rome, Italy; 5 Centre of Research on Health and Social Care Management, SDA Bocconi School of Management, Bocconi University, Milan, Italy; 6 Agency for Public Health, Lazio Region, Rome, Italy; 7 General Directorate of Health and Social Policies, Emilia-Romagna Region, Bologna, Italy; 8 Department of Health Services and Epidemiological Observatory, Regional Health Authority, Sicily Region, Palermo, Italy; 9 Center of Epidemiology and Biostatistics, Polytechnic University of Marche, Ancona, Italy; 10 Department of Mental Health and Addiction Services, ASST Lecco, Lecco, Italy

**Keywords:** Mental healthcare, Effective coverage, Self-controlled case series, Schizophrenic disorder, Real-world, Healthcare utilization database

## Abstract

**Purpose:**

To measure indicators of timeliness and continuity of treatments on patients with schizophrenic disorder in ‘real-life’ practice, and to validate them through their relationship with relapse occurrences.

**Methods:**

The target population was from four Italian regions overall covering 22 million beneficiaries of the NHS (37% of the entire Italian population). The cohort included 12,054 patients newly taken into care for schizophrenic disorder between January 2015 and June 2016. The self-controlled case series (SCCS) design was used to estimate the incidence rate ratio of relapse occurrences according to mental healthcare coverage.

**Results:**

Poor timeliness (82% and 33% of cohort members had not yet started treatment with psychosocial interventions and antipsychotic drug therapy within the first year after they were taken into care) and continuity (27% and 23% of patients were persistent with psychosocial interventions, and antipsychotic drug therapy within the first 2 years after starting the specific treatment) were observed. According to SCCS design, 4794 relapses occurred during 9430 PY (with incidence rate of 50.8 every 100 PY). Compared with periods not covered by mental healthcare, those covered by psychosocial intervention alone, antipsychotic drugs alone and by psychosocial intervention and antipsychotic drugs together were, respectively, associated with relapse rate reductions of 28% (95% CI 4–46%), 24% (17–30%) and 44% (32–53%).

**Conclusion:**

Healthcare administrative data may contribute to monitor and to assess the effectiveness of a mental health system. Persistent use of both psychosocial intervention and antipsychotic drugs reduces risk of severe relapse.

**Supplementary Information:**

The online version contains supplementary material available at 10.1007/s00127-021-02114-9.

## Introduction

In Italy, the transition from a hospital- to a community-based system started in 1978, with a reform that led to the gradual closing of psychiatric hospitals [1]. However, as the reform assigned the responsibility for managing the health system to regions, between-region heterogeneity in the quality of provided mental health care [2, 3] has become a main concern in the field.

Performance indicators are measurable elements of practice for which there is evidence for, or consensus on, their usefulness in assessing healthcare quality [4]. However, despite their widespread use [5–8], their validity (i.e., the ability of a process indicator to identify components of healthcare quality casually associated with clinical outcomes) is largely untested by randomized clinical trials [9], thus making their real-world evaluation essential.

Two multidisciplinary expert groups, both funded by the Italian Ministry of Health for Evaluating quality of care for severe mental disorders (QUADIM project, Health Prevention Department) and for Monitoring and assessing care pathways (MAP project [10], Health Planning Department), jointly designed a set of performance indicators for the assessment and comparison of mental healthcare quality among regions. However, as a better performance profile measured through these indicators is not necessarily associated with better outcomes, a validation study was designed. The current paper investigates the quality of mental healthcare delivered to patients newly taken into care for schizophrenic disorder by the National Health Service (NHS) of four Italian areas. Furthermore, the aim was to test the hypothesis that the higher the exposure to mental healthcare (i.e., timeliness and continuity of community treatments and appropriate drug therapy), the lower the likelihood that negative outcomes occur (i.e., hospital admission via emergency room, which we considered as a measurable surrogate of relapse occurrence).

## Methods

### Setting

In Italy, the access to physical (e.g., chronic conditions such as diabetes) and mental (e.g., schizophrenia) healthcare is provided according with health needs of beneficiaries of NHS. In particular, mental healthcare for patients with schizophrenic disorder is mainly provided by public Departments of Mental Health (DMHs), which are organized into a network of community services including Community Mental Health Centres, General Hospital Psychiatric Wards, Day-Care Centres, and Community Residential Facilities. The access for patients with schizophrenia to public DMHs is free and, according with our clinical experience, only few patients with diagnosis of schizophrenia receive care from private facilities (although, according with our best knowledge, it has never been measured).

### Data sources

The QUADIM project is based on computerized Healthcare Utilization (HCU) databases from three Italian regions: Lombardy (northwest Italy), Emilia-Romagna (northeast Italy) and Lazio (central Italy), and the province of Palermo (southern Italy). Overall, data covered almost 22 million beneficiaries of the Italian NHS, nearly 37% of the entire national population.

An automated system of HCU databases is used to manage health services within each region. HCU data include a variety of information on residents, such as diagnosis at discharge from public or private hospitals, outpatient drug prescriptions, specialist visits and diagnostic exams reimbursable by the NHS.

In addition, a specific automated system concerning psychiatric care gathers data from regional DMHs accredited by the NHS. This system, in addition to providing demographic information and diagnostic codes for patients receiving specialist mental healthcare, also collects information regarding the interventions and activities provided by DMHs on outpatient, home care or day care facilities. These interventions were coded and classified into two broad categories: “psychosocial interventions” and “generic care” (see the full list in Supplementary Table S1).

Because a unique identification code is used for all databases within each region, the complete care pathway of NHS beneficiaries can be obtained through record linkage. Further details on HCU database use in the field of mental health have been reported elsewhere [3, 9]. Diagnostic and drug therapy codes used for drawing records and fields from databases in the current study are reported in Supplementary Table S2.

The Ethical Committee of the University of Milano-Bicocca evaluated and approved the study protocol (Protocol number 497, Year 2019).

### Cohort selection

The target population consisted of all NHS beneficiaries resident in Lombardy, Emilia-Romagna, Lazio and Palermo, aged 18–65 years. According to the Italian Institute of Statistics, this population amounted to 13.5 million people in 2015 (http://demo.istat.it/index.html). The individuals who, during the recruitment period, had at least one contact with mental health services and had a diagnosis of schizophrenic disorder were identified. According to data availability, the following distinct recruitment periods were considered: from January 2013 to June 2016 for Lombardy, from January 2015 to June 2016 for Emilia-Romagna and Palermo district, and from January to December 2015 for Lazio. The patients were labeled as prevalent cases, and the date of their first contact with mental health services during the recruitment period was recorded as the index date.

With the aim of including individuals with first lifetime diagnosis of schizophrenic disorder known to the NHS, prevalent cases were excluded if they (1) had previously received a diagnosis of schizophrenic disorder at any time prior to the index date, (2) had experienced at least one hospital admission to a psychiatric ward, and/or (3) had received at least two prescriptions of antipsychotic drugs within 2 years before the index date. The remaining patients represented the study cohort and were labeled as patients newly taken into care for schizophrenic disorder.

Because there is some residual uncertainty regarding the ability of this algorithm to identify new diagnoses, the study cohort was restricted to patients aged 18–40 years in secondary analysis.

### Exposure to mental healthcare

The following three mental healthcare pathways, designed by the QUADIM/MAP working groups, were separately taken into account by checking the sequence of: (1) any outpatient contacts with mental health services resulting in at least one intervention among those listed in Supplementary Table S1 (hereafter referred to as “any outpatient care”); (2) any outpatient contacts with mental health services resulting in a psychosocial intervention (hereafter referred to as “psychosocial intervention”); (3) antipsychotic drugs directly dispensed by the mental health services or collected from a community pharmacy (hereafter referred to as “antipsychotic drug therapy”). For each pathway, the first occurrence of the specific contact with mental services was marked as care starting.

Each pathway was assessed investigating two dimensions of its specific performance indicator. The first measure adopted was the timeliness of starting treatment. In this case, the observation started from the index date and ended on the date of starting psychosocial intervention or antipsychotic therapy, or of censoring (occurring at death, migration, or twelve months after the index date). The cumulative proportion of cohort members who started treatment during the first year after they were taken into care was calculated using the Kaplan–Meier estimator.

The second measure we evaluated was the continuity of care. In this analysis, only cohort members who started treatment within the first year after they were taken into care were included. The observation began from the date of starting treatment (overlapping with index date for any outpatient care, or possibly delayed for psychosocial intervention and antipsychotic drug therapy) and ended at the first episode of discontinuation or censoring (death, migration, or study-end, i.e., December 31, 2016 for Lazio, and June 30, 2018 for the other regions). Discontinuation of any outpatient care or psychosocial intervention occurred when, for the first time, a patient did not receive care or interventions for 30 days or longer. Discontinuation from antipsychotic drug therapy occurred when, for the first time, the time span between the end of coverage following a given drug prescription and the next prescription was 30 days or longer (prescription coverage was calculated by dividing the total amount of the drug dispensed by the defined daily dose). The actuarial life-table method was used for calculating the month-by-month probability of continuing treatment (i.e., of not experiencing discontinuation) once it started.

### Outcome occurrence

Emergency hospital admissions to psychiatric wards that occurred during the observational period were recorded for each cohort member. The admissions reporting a diagnosis of mental disorder were labeled as outcome episodes and were considered as measurable surrogates of relapse [11, 12] (for this reason, we use the terms outcome and relapse interchangeably across the text).

### Mental healthcare and outcome association

To estimate the association between the exposure to the mental healthcare pathways above described and the risk of relapse occurrences (i.e., the validity of the performance indicators), we used a self-controlled case series (SCCS) design.

At first sight, a simple cohort design investigating the association between time-varying treatment exposure and relapse occurrence may be adopted. However, as relevant data, such as the severity of schizophrenia at cohort entry, comorbidities, and lifestyle factors (among others), cannot be measured in a study based on HCU data, a cohort approach likely generates estimates affected by between-person confounding. In other words, because patients affected by a more severe schizophrenia at baseline are more likely to receive timely and uninterrupted care, but also more frequently experience relapse episodes, a paradox positive association between intensity of mental healthcare and outcome occurrence may be generated from this simple design. For this reason, a within-patients self-controlled case series (SCCS) design was adopted [13]. SCCS is a case-only approach aimed at eliminating between-persons confounding. In our application, cohort members who experienced (1) both periods of exposure and non-exposure to mental healthcare (those who started and discontinued at least once care/treatment/therapy), and (2) at least one relapse episode, were included (see supplementary Figure S1, first scenario). For each included patient, the observational time window started on the index date and finished on the date of death, migration, or of study-end, whichever occurred earliest. The observational time window was then divided into subperiods accumulating person-months of coverage and non-coverage with specific mental healthcare. The exposure – outcome association was estimated using a conditional Poisson model to derive incidence rate ratios (IRR), comparing relapse rates occurred during mental healthcare coverage and non-coverage subperiods [14]. We also estimated the relapse episodes that were potentially avoidable by ensuring whole coverage with mental healthcare. Quantities considered for this analysis were the IRR associated with, and person-years (PY) covered by, the considered categories of mental healthcare, e.g. (1) neither any outpatient care or psychosocial intervention nor antipsychotic drug therapy; (2) any outpatient care or psychosocial intervention only, (3) antipsychotic drug therapy only, and (4) both any outpatient care or psychosocial intervention and antipsychotic drug therapy.

In addition to unmeasured confounding, we also reasoned about three other potential sources of bias [15]. First, as increasing use of mental healthcare is expected just before the relapse occurrence because of worsening symptoms, with the aim of avoiding the subsequent protopathic bias [16], a lag-time of 30 days before the outcome was removed [17] (see supplementary Figure S1, second scenario). Second, because one key assumption of SCCS is that the outcome occurrence should not alter the probability of subsequent exposure [13], a 30-day period was also removed after hospital discharge (see supplementary Figure S1, third scenario). Finally, because another key assumption of the SCCS approach is that relapse episodes must be independent (i.e., the likelihood of the occurrence of a relapse episode must be not influenced by having experienced previous relapses), only the first outcome was considered [18], i.e., observational period was censored when the first relapse occurred (see supplementary Figure S1, fourth scenario). The most precautionary design towards possible biases that at the same time uses all available data (i.e., that shown in Figure S1, third scenario) was considered in the main analyses, while the other designs were taken into account for checking the robustness of the main results. In addition, due to arbitrariness in the choice of the time-frame length before and after relapse occurrence, the robustness of the main findings was tested also by varying the sizes.

### Between-region summarized estimates

Since the recent privacy regulations limit the free movement of electronic health data, pseudo-anonymized data were extracted and processed locally using a communal Statistical Analysis System (SAS) program, developed by one author (MMC) according to the protocol previously shared and approved by the QUADIM/MAP working groups. Thus, the above calculations were separately performed within each considered region/province and summarized estimates were obtained by pooling aggregated regional data.

Summarized Kaplan–Meier survival curves picturing the timeliness of starting a specific treatment, were obtained by a method for building individual patient data starting from region curves. Briefly, a digital software was used to read the coordinates of the survival curves in each region. Information on the number of patients still at risk at each month of follow-up and on the total number of discontinuation events was used to solve the inverted survival equation, therefore obtaining pooled individual-patient data [19].

Instead, regarding the association between the exposure to mental healthcare and the risk of relapse occurrences, the approach proposed by DerSimonian and Laird was used for summarizing between-region IRRs [20]. The heterogeneity of estimates between regions was tested with Cochran’s *Q* test and measured with the *I*^2^ statistics (the proportion of between-region variability due to heterogeneity) [21].

The Statistical Analysis System Software (version 9.4; SAS Institute, Cary, NC, USA) was used to perform the analyses. For all hypotheses tested, two-tailed *p* values less than 0.05 or, in an equivalent manner, 95% CI of OR that does not contain the value expected under the null hypothesis, were considered significant.

### Role of the funding source

The study’s sponsor, i.e., Italian Ministry of Health, had no role in the design of the study, the collection, analysis, and interpretation of the data or in the writing of the manuscript.

## Results

### Patients

The process of cohort selection is shown in Figure S2. Among the 75,233 eligible prevalent cases, 63,179 were excluded (mostly because of a previous diagnosis of schizophrenic disorder), while 12,054 individuals met the inclusion criteria and were enrolled into the study cohort as newly taken-into-care patients. The diagnostic codes distribution used for selecting the entire cohort of first contact patients, as well as the cohort portion aged 40 years or younger at diagnosis, is shown in Supplementary Table S3. Raw prevalence rates of treated schizophrenic disorder per 1000 inhabitants ranged between 2.4 (Lazio) and 5.2 (Lombardy), with an overall prevalence rate of 4.2. Age-standardized rates of patients newly taken-into-care for schizophrenic disorder ranged between 2.0 (Lombardy) and 3.8 (Palermo) per 10,000 PY (overall incidence of 3.1).

The baseline characteristics of the 12,054 newly taken-into-care cohort members are shown in Table 1. The mean age was about 45 years and 55% of patients were men. Most patients had low education (about 61% had less than 8 years of education) and almost 50% of them were unemployed.

**Table 1:** Baseline characteristics of patients newly taken into care for schizophrenia in four Italian areas (Lombardy, Emilia Romagna and Lazio Regions and Province of Palermo) and in the whole sample. Italy, QUADIM Project, 2013–2018

	Lombardy (*N* = 7004)	Emilia-Romagna (*N* = 2121)	Lazio (*N* = 2209)	Palermo (*N* = 720)	All together (N = 12,054)
Gender					
Men	3790 (54.1%)	1191 (56.2%)	1163 (52.6%)	463 (64.3%)	6,607 (54.8%)
Women	3214 (45.9%)	930 (43.8%)	1046 (47.4%)	257 (35.7%)	5447 (45.2%)
Age (years)					
Mean (SD)	41.6 (13.2)	42.2 (12.6)	42.6 (12.2)	50.5 (15.6)	44.5 (13.4)
18–30	1639 (23.4%)	478 (22.5%)	268 (12.1%)	148 (20.6%)	2533 (21.0%)
30–39	1104 (15.8%)	374 (17.6%)	278 (12.6%)	135 (18.8%)	1891 (15.7%)
40–49	1496 (21.4%)	590 (27.8%)	519 (23.5%)	195 (27.0%)	2800 (23.2%)
50–64	2765 (39.4%)	679 (32.1%)	1144 (51.8%)	242 (33.6%)	4830 (40.1%)
Years of education					
0–5	2354 (33.6%)	208 (9.8%)	353 (16.0%)	159 (22.1%)	3074 (25.5%)
6–8	2190 (31.3%)	784 (37.0%)	846 (38.3%)	515 (71.5%)	4335 (36.0%)
9–13	1468 (21.0%)	670 (31.6%)	611 (27.7%)	0 (0%)	2749 (22.8%)
≥ 14	346 (4.9%)	169 (8.0%)	128 (5.8%)	32 (4.5%)	675 (5.6%)
* Missing data*	646 (9.2%)	290 (13.6%)	271 (12.2%)	14 (1.9%)	1221 (10.1%)
Employment status					
Employed	2803 (40.0%)	520 (24.5%)	475 (21.5%)	105 (14.6%)	3903 (32.4%)
Unemployed	2693 (38.4%)	998 (47.1%)	1506 (68.2%)	554 (76.9%)	5751 (47.7%)
Retired	942 (13.4%)	148 (7.0%)	2 (0.1%)	44 (6.1%)	1136 (9.4%)
* Missing data*	566 (8.2%)	455 (21.4%)	226 (10.2%)	17 (2.4%)	1264 (10.5%)
Living arrangements					
With family	5155 (73.6%)	1494 (70.4%)	NA	244 (33.9%)	6893 (70.0%)
In residential facility	301 (4.3%)	102 (4.8%)	NA	53 (7.4%)	456 (4.6%)
Alone	1063 (15.2%)	243 (11.5%)	NA	51 (7.1%)	1357 (13.8%)
*Missing data*	485 (6.9%)	282 (13.3%)	NA	372 (51.6%)	1139 (11.6%)
Marital status					
Unmarried	3708 (52.9%)	1279 (60.3%)	1318 (59.7%)	473 (65.7%)	6778 (56.2%)
Married	1966 (28.1%)	433 (20.4%)	491 (22.2%)	163 (22.6%)	3053 (25.3%)
Separated	320 (4.6%)	80 (3.8%)	80 (3.6%)	11 (1.5%)	491 (4.1%)
Divorced	233 (3.3%)	65 (3.1%)	108 (4.9%)	34 (4.7%)	440 (3.7%)
Widowed	323 (4.6%)	29 (1.4%)	58 (2.6%)	7 (1.0%)	417 (3.5%)
*Missing data*	454 (6.5%)	235 (11.0%)	154 (7.0%)	32 (4.5%)	875 (7.2%)
Multisource Comorbidity Score					
0	2914 (41.6%)	1258 (59.3%)	1516 (68.6%)	411 (57.1%)	6099 (50.6%)
1–5	957 (13.7%)	362 (17.1%)	190 (8.6%)	89 (12.4%)	1598 (13.3%)
6–10	2545 (36.3%)	409 (19.3%)	393 (17.8%)	178 (24.6%)	3525 (29.2%)
11–15	362 (5.2%)	56 (2.6%)	70 (3.2%)	25 (3.5%)	513 (4.3%)
≥ 16	226 (3.2%)	36 (1.7%)	40 (1.8%)	17 (2.4%)	319 (2.6%)

### Exposure to mental healthcare

**Fig. 1:**
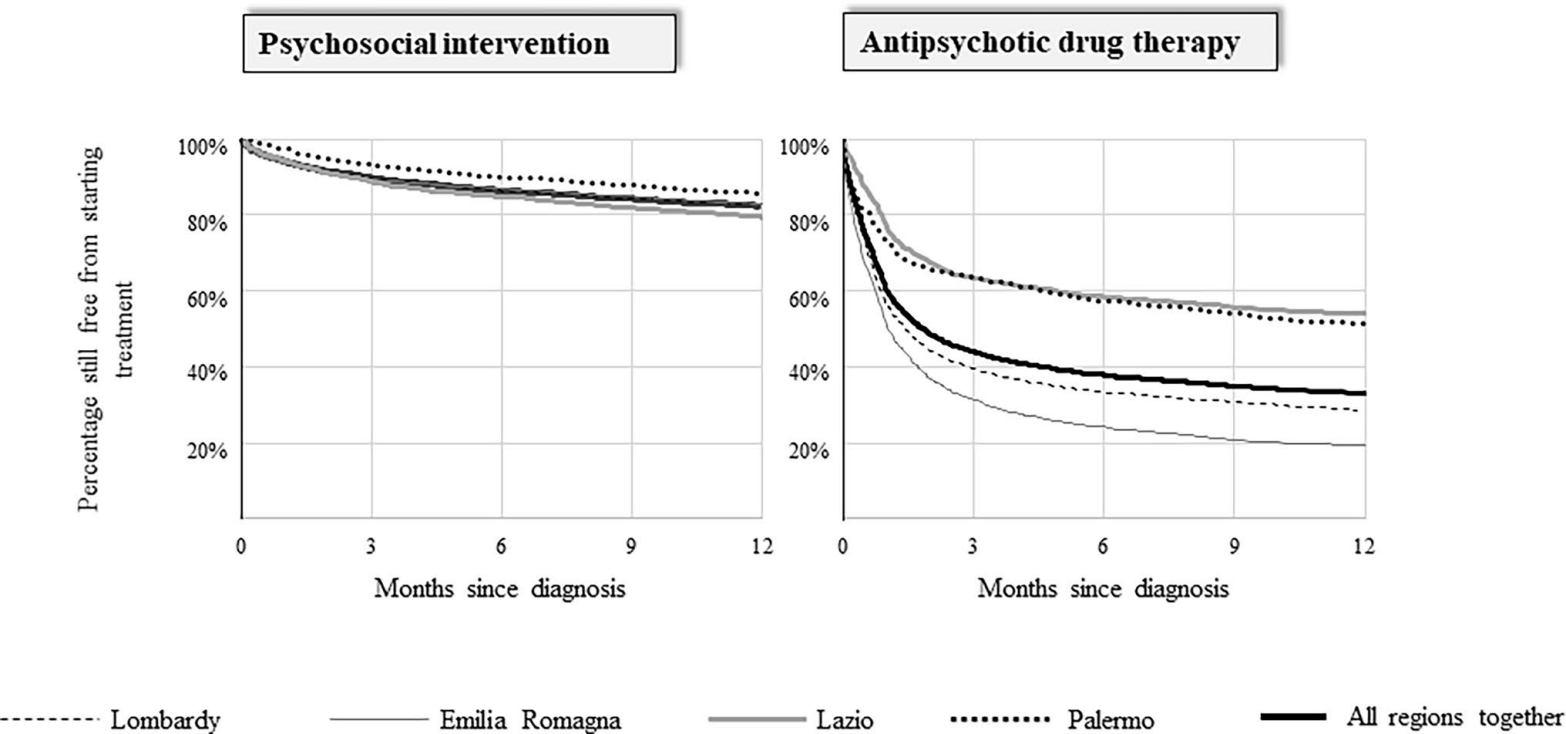
Timeliness of starting psychosocial intervention and antipsychotic drug therapy in three regions (Lombardy, Emilia Romagna, and Lazio) and one province (Palermo), and in the whole Italian sample. Italy, QUADIM-MAP projects, Italy, 2013–2018. The observation started from the index date and ended on the date of starting therapy; the cumulative proportion of cohort members who started therapy during the first year after they were taken into care was calculated through the Kaplan–Meier estimator

Poor timeliness of starting mental healthcare was observed. Within the first year after intake, 82% and 33% of cohort members had not yet started treatment with psychosocial interventions and antipsychotic drug therapy, respectively (Fig. 1). Between-region variability of psychosocial interventions was low. Conversely, there was a large between-region heterogeneity for starting drug therapy, with the proportion of patients still free from therapy ranging between 19% (Emilia-Romagna) and 54% (Lazio).

**Fig. 2:**
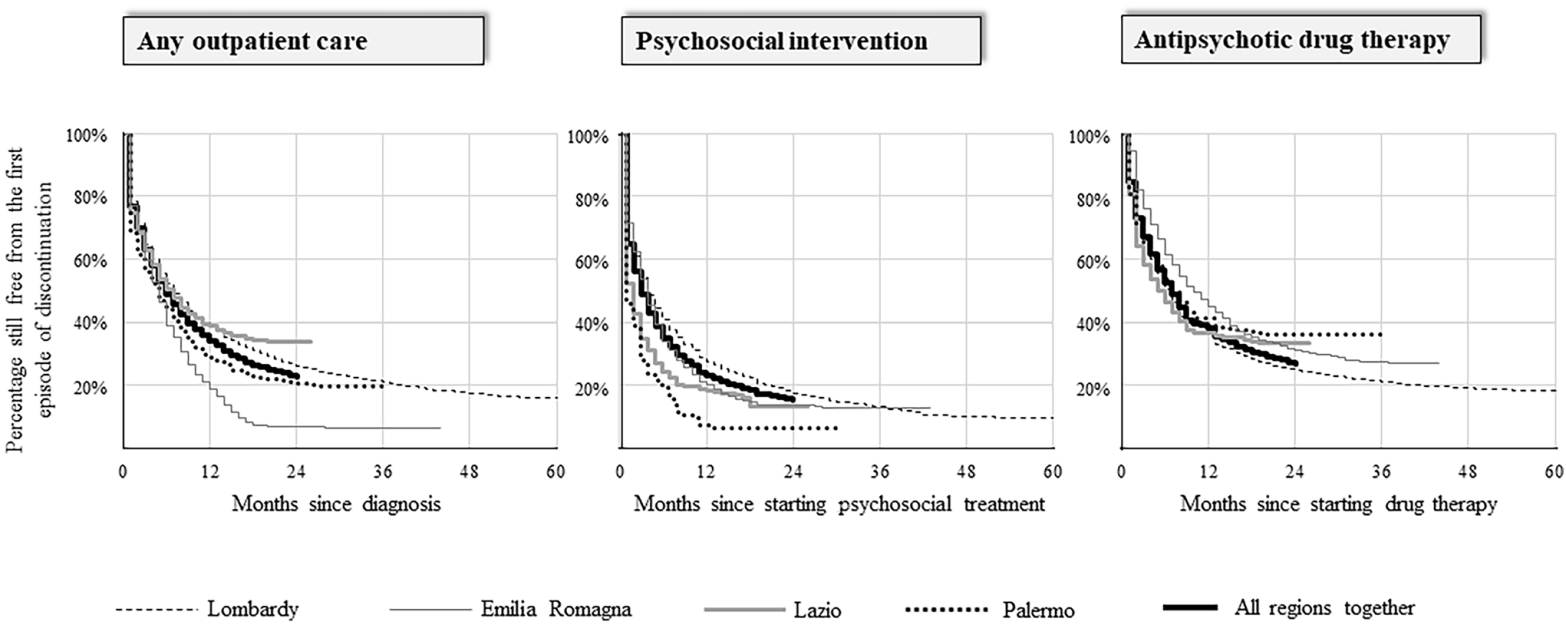
Probability of continuing any outpatient care, psychosocial intervention, and antipsychotic drug therapy in three regions (Lombardy, Emilia Romagna, and Lazio) and one province (Palermo), and in the whole Italian sample. Italy, QUADIM-MAP projects, Italy, 2013–2018. Cohort members who started therapy within the first year after they were taken into care were included; the observation started from the date of starting therapy and ended at the occurrence of the first episode of treatment discontinuation; the actuarial life-table method was used for calculating month-by-month probability of continuing therapy (i.e., of not experiencing discontinuation) once it started

Continuity of contacts with mental health services providing any outpatient care, psychosocial interventions, and antipsychotic drug therapy within 2 years after starting the same intervention concerned 23%, 15%, and 27% of patients, respectively (Fig. 2). Large between-region heterogeneity for continuity of care was observed, with the two-year proportion of patients continuing care ranging between 7 (Emilia-Romagna) and 34% (Lazio) for any outpatient care, 6% (Palermo) and 19% (Lombardy) for psychosocial interventions, and between 25 (Lombardy) and 38% (Palermo) for antipsychotic drug therapy.

### Outcome occurrence

On the whole, cohort members accumulated 36,008 PY of follow-up and generated 6592 relapse episodes, with an overall incidence rate of 18.3 every 100 PY (ranging from 8.8 to 21.6 every 100 PY in Palermo and Emilia-Romagna, respectively).

### Mental healthcare and outcome association

**Fig. 3:**
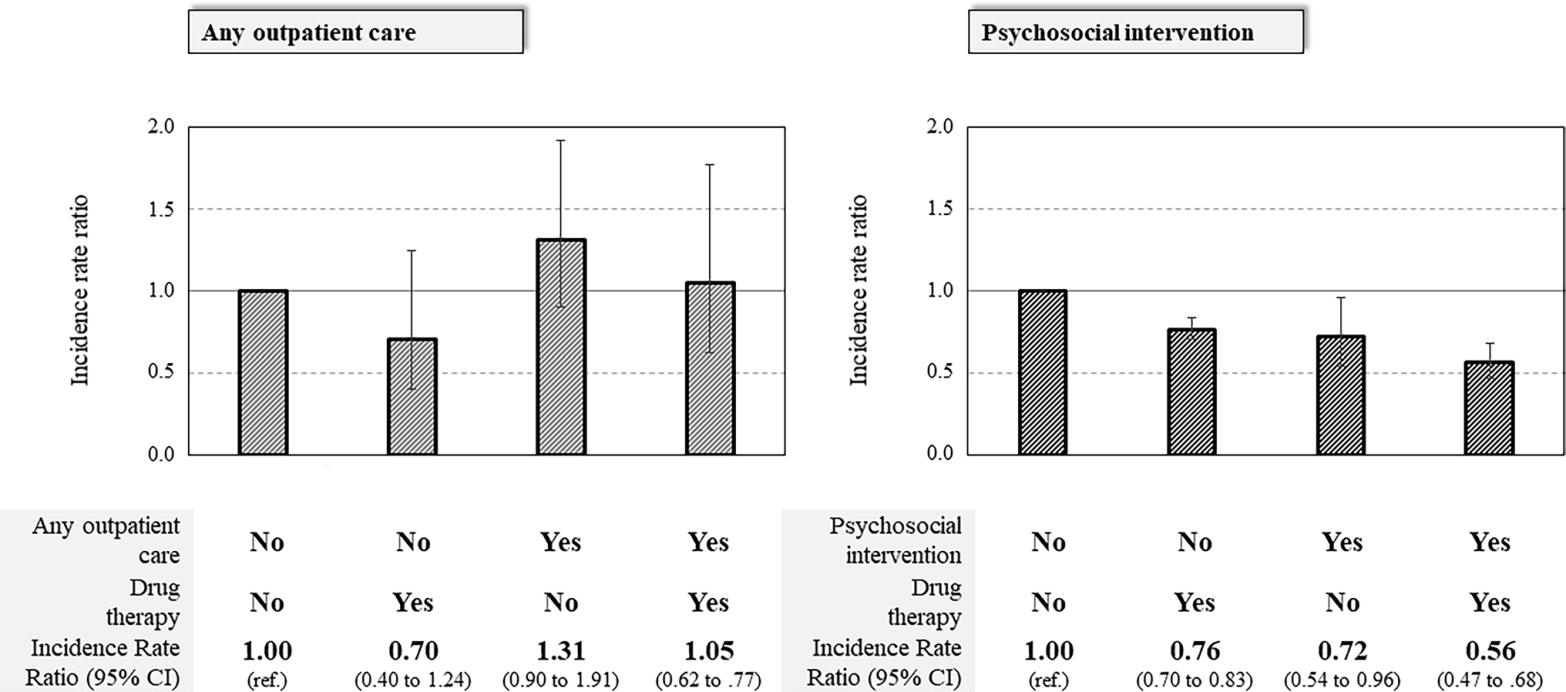
Summarized self-controlled case series estimates of the incidence rate ratio of emergency mental health-related hospital admissions associated with any outpatient care and antipsychotic drug therapy (left box) and psychosocial intervention and antipsychotic drug therapy (right box). Italy, QUADIM-MAP projects, Italy, 2013–2018. Self-controlled case series incidence rate ratio, and 95% confidence interval, estimated with conditional Poisson regression contrasting within-patient relapse incidence during time windows of coverage and no coverage with mental health care. Estimates were obtained through the design shown in supplementary Figure S1, third scenario

According to the SCCS design, a total of 4794 relapses occurred during 9430 PY (with an incidence rate of 50.8 every 100 PY). Models investigating any outpatient care (Fig. 3, left box) did not offer evidence of association between any outpatient care and relapse incidence. Conversely, models including psychosocial interventions (Fig. 3, right box) showed that, compared with the relapse rate during periods not covered by either psychosocial intervention or antipsychotic drug therapy (54.9 every 100 PY), those covered by psychosocial intervention alone, by antipsychotic drug therapy alone and by psychosocial intervention and antipsychotic drug therapy together were, respectively, associated with relapse rate reductions of 28% (95% CI 4–46%), 24% (95% CI 17–30%) and 44% (95% CI 32–53%). A reduction in the incidence of relapse from 50.8 to 17.0 per 100 PY would have been observed if the observation period had been entirely covered by both psychosocial intervention and antipsychotic drug therapy, with a percentage of preventable relapse of 66%.

There was no evidence of between-region heterogeneity related to such effects (supplementary Table S4). The relationships described above did not substantially change by (1) adopting less stringent criteria in designing the SCCS, (2) varying the sizes of time windows in the SCCS design (supplementary Table S5), and (3) restricting the cohort to patients aged 40 years or younger (supplementary Table S6).

## Discussion

The current paper investigated the exposure to two components of the quality of care delivered to patients newly taken-into-care by the NHS for schizophrenic disorder (nominally timeliness and continuity of mental healthcare) and tested the hypothesis that the higher the exposure to this healthcare, the lower the likelihood that negative outcomes occur.

As far as timeliness and continuity, we assumed that both antipsychotic drug therapy and outpatient care should start as soon as possible after diagnosis, and once started, mental healthcare services should be kept with a frequency of one intervention every month, discontinuation was assumed otherwise. Nevertheless, about one in three of the 12,054 newly taken-into-care patients with a diagnosis of schizophrenic disorder included in this population-based investigation had not started yet an antipsychotic drug therapy in the first year after diagnosis. Moreover, once started, antipsychotic drug therapy was frequently discontinued, with only 27% patients being persistent with drug therapy for at least 2 years after diagnosis. In addition, only 23% of patients did not discontinue any outpatient care within 2 years after diagnosis. These findings were widely expected, since a number of studies report that treatment is frequently discontinued by patients with schizophrenia disorder ^[34–37]^, even in the Italian setting [14, 15, 21].

Between-region heterogeneity mostly regarded timeliness of starting drug therapy and continuity of outpatient care. Regional variations in the management of schizophrenia are likely due to local differences in the amount and allocation of public resources employed for mental health care [24]. Future research should investigate the organizational characteristics of local services (including human and financial resources) that can deliver effective mental healthcare, minimizing the risk of relapse, with the lowest healthcare cost.

The new important findings, however, are that (1) only 18% of cohort members received psychosocial intervention at least once in the first year after intake; (2) psychosocial intervention was frequently discontinued, with only 15% continuing treatment for least 2 years after starting; (3) a clear reduction in relapse occurrence during periods covered by psychosocial interventions was observed, while the same did not happen when exposure to any outpatient care was considered. The effect of healthcare coverage was not trivial, as we found that 66% of relapses could have been avoided if the entire observation period had been covered by psychosocial and pharmacological treatments together.

In agreement with the landmark paper of Tanahashi [22], the concepts of contact coverage and effective coverage were fully supported by our findings. In fact, our study showed that contact coverage, i.e., timeliness of starting any outpatient care, such as attending services monthly, is not sufficient for protecting the patient from relapse occurrence. Conversely, effective coverage, i.e., ensuring that the patient promptly starts (and regularly receives) psychosocial interventions and antipsychotic drug therapy, showed evidence of protection from relapse occurrence. This means that, consistently with Pathare and colleagues [23], a comprehensive measure of mental healthcare gap for persons with severe mental illness, including schizophrenia disorder, should take into account not only ‘treatment gap’, as currently understood and measured, but also ‘psychosocial intervention gap’, i.e., the lack of psychosocial interventions. Our study shows that currently, in Italy, only a small portion of the patients taken into care by mental health services receive effective care (i.e., uninterrupted treatment with psychosocial care or antipsychotic drug therapy). This indicator(s) may be useful to assess to what extent a national mental health system is effective.

The present study is unique in several respects. First, the investigation is based on data from a large unselected population, which was possible because in Italy, a free healthcare system is available to all citizens. In particular, the availability of high-quality individual data on outpatient and inpatient services provided by the NHS, which can be linked to data on care provided by mental health departments in the last 10 years or so (retrieved from the so-called mental health information system), offers the opportunity to design investigations including large unselected populations, and to generate real-world evidence on mental health care [3, 9, 25, 26]. Second, our data reflect routine clinical practice, and are not affected by selective participation and recall bias. Third, patients were identified from the day of the first contact with the NHS mental health services in which a schizophrenic disorder was diagnosed, and the complete sequence of mental healthcare services was known. Fourth, because (to the best of our knowledge) the SCCS design has never been used previously to measure the effectiveness of mental healthcare, our approach provides an original methodological hint for further research in this field. Finally, a number of sensitivity analyses confirmed the robustness of our findings.

To understand our results correctly, limitations of this study should also be taken into account. First, using HCU databases, we were reasonably able to detect only the first contact with diagnosis of schizophrenic disorder that was registered with the public Regional Health System, and not the date of the real onset of this mental disorder. Indeed, although we designed our study to start observation from the point of time when a patient was diagnosed with schizophrenic disorder, our findings suggest that this did not always occur. In fact, as observation started after 40 years of age for 63.3% of the cohort members, we suspect that a diagnosis already existed for several patients, since the onset of schizophrenic disorder is usually less likely to occur after the age of 40 years. This is likely due to our inability to account for private services and should underline that our findings only focus on services supplied by public facilities. However, in a specific sensitivity analysis, we showed that, restricting the cohort to patients aged 40 years or younger, the protective effect of continuity of psychosocial intervention and antipsychotic drug therapy on the occurrence of relapse was still observed (supplementary Table S6), indeed being even more marked than in the main analysis, thus strengthening the robustness of the main findings of our study.

Second, we cannot exclude that some between-region differences are in part due to heterogeneity in quality and completeness of data. However, it should be stressed that, because healthcare utilization data are used for reimbursing public and accredited service providers, incorrect and incomplete reports lead to legal consequences.

Third, both exposure and outcome misclassification likely affected our estimates. Common sources of exposure misclassification include treatments delivered by private services or office-based practices, as well as out-of-pocket payments for healthcare services [27, 28]. However, since misclassification of mental healthcare coverage likely occurs irrespectively from experiencing relapse (i.e., being likely that a no-differential misclassification occurs [29]), unbiased estimates are expected. Outcome misclassification may be due to our inability to capture all relapse episodes, but only the most severe requiring hospital admissions. Thus, we highlight that our findings only concern more severe forms of relapse and our conclusions only refer to mental healthcare able of preventing detectable relapse episodes (e.g., the most severe cases, requiring hospitalization). On the other hand, we cannot exclude the possibility that generic care may be effective for preventing relapses in milder cases.

Finally, a limited amount of available clinical information engenders confounding. In fact, as patients with frequent contacts are expected to have more severe clinical manifestations than those with fewer contacts, common observational designs could not directly account for confounders. However, as a SCCS (within-patient) design [13, 14] was used in our study, confounding by time-invariant attributes does not affect our estimates. Moreover, the effect of the sudden worsening of symptoms (i.e., the protopathic bias) has been taken into account in our study through a lag-time approach [17]. Finally, although our research question potentially violates some of the assumptions of the self-controlled case series methodology, we used recommended approaches to address these issues, including studying only first events and including pre- and post-outcome periods in the analyses.

## Conclusion

Our study confirms that administrative data may contribute to assessing the effectiveness of a mental health system even in the absence of ad hoc data collection. It also showed that psychosocial interventions should be considered as the “core” of community care (i.e., the most protective from the risk of experiencing severe relapse) and should be increased in terms of intensity and continuity in community mental health services, particularly for newly taken-into-care patients. Finally, our investigation offered some evidence that the persistent use of both psychosocial interventions by community mental health services and antipsychotic drug therapy reduces the risk of severe relapse in people with schizophrenia spectrum disorders. Better information about mental healthcare effective coverage is essential.

## Supplementary Information

Below is the link to the electronic supplementary material.

Supplementary file1 (DOCX 38 KB)

Supplementary file2 (TIF 128 KB)

Supplementary file3 (TIF 117 KB)

## Data Availability

The data that support the findings of this study are available from the Regions of Lombardy, Lazio and Emilia-Romagna, and the Province of Palermo, but restrictions apply to the availability of these data, which were used under license for the current study, and so are not publicly available. Data are however available from the authors upon reasonable request and with permission of the Regions involved in this study.
