# Supplementary material for: Does the mental health system provide effective coverage to people with schizophrenic disorder? A self-controlled case series study in Italy
Source: Soc Psychiatry Psychiatr Epidemiol. 2021 Jun 16;57(3):519–29. doi: 10.1007/s00127-021-02114-9 (PMC8934324; doi:10.1007/s00127-021-02114-9)
Supplement: Supplementary file 1 — Supplementary file1 (DOCX 38 KB) [file 127_2021_2114_MOESM1_ESM.docx]

### Supplementary material

**Legends of supplementary Figures**

**eFigure S1.** Graphical representation of framework for Self-Controlled Case Series for a cohort member

**Footnote**. Cohort members who experienced both exposure to mental health care and at least a relapse episode were included. Observational time-window started at the index date (i.e., the patients was newly taken in care with diagnosis of schizophrenic disorder) and finished at the date of death, emigration, or of study-end whichever occurred earliest. Observational time-window was portioned in subperiods of coverage and no coverage with mental health care. Incidence rate ratio (IRR) was then derived comparing the relapse rate accumulated during coverage and no coverage person-month. In the first scenario, one relapse event occurred during eight person-month of coverage, and another one during four person-month of no coverage, being the corresponding IRR=0.5. In the second scenario, with the aim of avoiding protopathic bias, a time-window of thirty days was removed before relapse occurring, so dragging incidence rates to 1/7 and 1/3 and the corresponding IRR=0.43. In the third scenario, with the aim of correcting for the modified likelihood after the relapse occurred, a time-window of thirty days was removed after each hospital discharge, so dragging incidence rates to 1/7 and 1/1 and the corresponding IRR=0.14. Finally, in the fourth scenario, with the aim of correcting for the lack of between-event independence, only the first relapse event occurred during the observational period was considered, so dragging incidence rates to 1/3 and 0/1 and the corresponding IRR=∞.

**eFigure S2**. Flow-chart of inclusion and exclusion criteria in three regions (Lombardy, Emilia Romagna and Lazio) and one province (Palermo), and in the whole Italian sample. Italy, QUADIM-MAP projects, Italy, 2013-2018

**Supplementary eTable 1.** Service interventions and activities classified in the Mental Health Information System

| **OUTPATIENT VISITS** (delivered by territorial and day-care facilities of CMHCs) | **Italian Mental Health Information system codes** |
| --- | --- |
| **Generic care** |  |
| Psychiatric visit | 01 |
| Clinical psychological interview | 02 |
| Interviews | 03 |
| Consultation | 04 |
| Medical-legal assessment | 05 |
| Standardized psychological assessments using test | 06 |
| Individual psychotherapy **†** | 07 |
| Couple psychotherapy **†** | 08 |
| Family psychotherapy **†** | 09 |
| Group psychotherapy **†** | 10 |
| Drugs administration | 11 |
| Interview with family members | 12 |
| Single family psychoeducation **†** | 13 |
| Multifamily group psychoeducation **†** | 14 |
| Staff Meeting | 15 |
| Individual living skills training **†** | 16 |
| Group living skills training **†** | 17 |
| Individual socialization intervention **†** | 18 |
| Group socialization intervention **†** | 19 |
| Healthcare facilities stay | 20 |
| Individual bodywork (i.e., expressive, practical manual and motor intervention) **†** | 21 |
| Group bodywork (i.e., expressive, practical manual and motor intervention) **†** | 22 |
| Work training **†** | 23 |
| Support interventions | 24 |
| Interventions for financial and welfare procedures | 25 |
| Network interventions | 26 |
|  |  |

**†** Outpatient contacts with mental health services resulting in a psychosocial intervention.

**Supplementary eTable 2.** Diagnostic and therapeutic (ICD-9-CM, ICD-10 and ATC) codes used in the current study for drawing records and fields from Healthcare Utilization databases

| **Schizophrenic spectrum disorders** | **ICD-10 codes**  **(Lombardy)** |
| --- | --- |
| Schizophrenia | F20.* |
| Schizotypal disorder | F21.* |
| Delusional disorders | F22.* |
| Brief psychotic disorder | F23.* |
| Shared psychotic disorder | F24.* |
| Schizoaffective disorders | F25.* |
| Other psychotic disorder not due to a substance or known physiological condition | F28.* |
| Unspecified psychosis not due to a substance or known physiological condition | F29.* |
|  | **ICD-9-CM codes**  **(Emilia-Romagna, Lazio and Palermo)** |
| Schizophrenic disorders | 295.* |
| Delusional disorders | 297.* |
| Other nonorganic psychoses | 298.2, 298.3, 298.8, 298.9 |
| Psychogenic paranoid psychosis | 298.4 |
| **Drugs** | **ATC codes** |
| Antipsychotic agents | N05A (excluded N05AN) |

**Supplementary eTable 3**. Distribution of ICD-10 (Lombardy) and ICD-9 (Emilia-Romagna, Lazio and Palermo) diagnostic codes used in the current study for selecting patient with schizophrenic spectrum disorder. All the included newly taken in care patients and those younger than 40 years are considered.

**Lombardy**

| **Schizophrenic spectrum disorder (ICD-10 Diagnostic Code)** | **All** | **Younger** |
| --- | --- | --- |
| Schizophrenia (F20.*) | 1,514 (21.6%) | 602 (20.7%) |
| Schizotypal disorder (F21.*) | 214 (3.1%) | 110 (3.8%) |
| Delusional disorders (F22.*) | 1,042 (14.9%) | 234 (8.0%) |
| Brief psychotic disorder (F23.*) | 1,383 (19.8%) | 732 (25.1%) |
| Shared psychotic disorder (F24.*) | 24 (0.3%) | 14 (0.5%) |
| Schizoaffective disorders (F25.*) | 493 (7.0%) | 163 (5.6%) |
| Other psychotic disorder not due to a substance or known physiological condition (F28.*) | 102 (1.5%) | 42 (1.4%) |
| Unspecified psychosis not due to a substance or known physiological condition (F29.*) | 1,430 (20.4%) | 690 (23.7%) |
| Two digit code (F2.*) | 802 (11.5%) | 327 (11.2%) |
| Total | 7,004 | 2,914 |

**Emilia-Romagna, Lazio and Palermo**

| **Psychoses (ICD-9 Diagnostic Code)** | **Emilia-Romagna** | | **Lazio** | | **Palermo** | |
| --- | --- | --- | --- | --- | --- | --- |
|  | **All** | **Younger** | **All** | **Younger** | **All** | **Younger** |
| Schizophrenic disorders (295.*) | 1,441 (65.2%) | 313 (53.9%) | 462 (64.2%) | 157 (52.5%) | 1,004 (47.3%) | 393 (43.3%) |
| Delusional disorders (297.*) | 268 (12.1%) | 65 (11.2%) | 40 (5.6%) | 18 (6.0%) | 542 (25.6%) | 178 (19.6%) |
| Other nonorganic psychoses (298.*, excluding 298.4) | 432 (19.5%) | 184 (31.7%) | 213 (29.5%) | 121 (40.5%) | 522 (24.6%) | 314 (34.6%) |
| Psychogenic paranoid psychosis (298.4) | 68 (3.1%) | 19 (3.3%) | 5 (0.7%) | 3 (1.0%) | 53 (2.5%) | 23 (2.5%) |
| Total | 2,209 | 581 | 720 | 299 | 2,121 | 908 |

**Supplementary eTable 4.** Region-specific self-controlled case series estimates of the incidence rate ratio of emergency mental health-related admissions associated with coverage with any outpatient care and antipsychotic drug therapy (left columns) and psychosocial intervention and antipsychotic drug therapy (right columns). Italy, QUADIM-MAP projects, Italy, 2013-2018

|  |  | **Lombardy** | | **Emilia Romagna** | |
| --- | --- | --- | --- | --- | --- |
| **Outpatient care** | **Drug therapy** | **Any outpatient care** | **Psychosocial intervention** | **Any outpatient care** | **Psychosocial intervention** |
| No | No | 1.00 (Ref.) | 1.00 (Ref.) | 1.00 (Ref.) | 1.00 (Ref.) |
| No | Yes | 0.71 (0.61 to 0.84) | 0.78 (0.71 to 0.85) | 1.52 (1.24 to 1.85) | 0.86 (0.35 to 2.11) |
| Yes | No | 1.76 (1.60 to 1.94) | 0.79 (0.64 to 0.98) | 0.86 (0.66 to 1.13) | 0.83 (0.55 to 1.24) |
| Yes | Yes | 1.08 (0.97 to 1.21) | 0.58 (0.48 to 0.71) | 1.91 (1.50 to 2.42) | 0.62 (0.30 to 1.28) |
|  |  | **Lazio** | | **Palermo** | |
|  |  | **Any outpatient care** | **Psychosocial intervention** | **Any outpatient care** | **Psychosocial intervention** |
| No | No | 1.00 (Ref.) | 1.00 (Ref.) | 1.00 (Ref.) | 1.00 (Ref.) |
| No | Yes | 0.29 (0.16 to 0.53) | 0.19 (0.2 to 0.29) | 0.64 (0.40 to 1.01) | 0.96 (0.69 to 1.34) |
| Yes | No | 1.60 (1.09 to 2.35) | 0.35 (0.17 to 0.74) | 1.18 (0.82 to 1.69) | 0.74 (0.22 to 2.47) |
| Yes | Yes | 0.32 (0.9 to 0.52) | 0.19 (0.06 to 0.54) | 1.60 (1.04 to 2.47) | 0.34 (0.04 to 2.69) |

**Footnote**. Self-controlled case series incidence rate ratio, and 95% confidence interval, estimated with Poisson regression contrasting within patient relapse incidence during time window of exposure and non-exposure to mental health care. Estimates were obtained through the design portrayed in supplementary **Figure S1**, third scenario

**Supplementary eTable 5.** Sensitivity analyses reporting estimates of Incidence Rate Ratios obtained from self-controlled case series scenarios (see Figure S1) and by varying time-windows widths of self-controlled case series design. Italy, QUADIM-MAP projects, Italy, 2013-2018

|  |  | **First scenario** | |
| --- | --- | --- | --- |
| **Outpatient care** | **Drug therapy** | **Any outpatient care** | **Psychosocial intervention** |
| No | No | 1.00 (Ref.) | 1.00 (Ref.) |
| No | Yes | 0.77 (0.51 to 1.17) | 0.74 (0.42 to 1.31) |
| Yes | No | 1.36 (0.96 to 1.94) | 0.88 (0.76 to 1.03) |
| Yes | Yes | 1.14 (0.75 to 1.74) | 0.78 (0.68 to 0.89) |
|  |  | **Second scenario** | |
|  |  | **Any outpatient care** | **Psychosocial intervention** |
| No | No | 1.00 (Ref.) | 1.00 (Ref.) |
| No | Yes | 0.68 (0.57 to 0.80) | 0.77 (0.70 to 0.84) |
| Yes | No | 1.75 (1.59 to 1.92) | 0.81 (0.65 to 1.00) |
| Yes | Yes | 1.10 (0.99 to 1.22) | 0.66 (0.55 to 0.79) |
|  |  | **Fourth scenario** | |
|  |  | **Any outpatient care** | **Psychosocial intervention** |
| No | No | 1.00 (Ref.) | 1.00 (Ref.) |
| No | Yes | 0.73 (0.45 to 1.21) | 0.70 (0.38 to 1.30) |
| Yes | No | 1.32 (0.89 to 1.94) | 0.79 (0.67 to 0.93) |
| Yes | Yes | 1.08 (0.67 to 1.75) | 0.68 (0.58 to 0.81) |

See footnote of **Figure S1** and **Figure 3** for further details

**Supplementary eTable 6.** Summarized self-controlled case series estimates of the incidence rate ratio of relapse associated with coverage with any outpatient care and antipsychotic drug therapy (left columns) and psychosocial intervention and antipsychotic drug therapy (right columns). Cohort members aged 40 years or younger were considered in this analysis (the corresponding data for the entire cohort being presented in Figure 3). Italy, QUADIM-MAP projects, Italy, 2013-2018

| Outpatient care | Drug therapy | Any outpatient care | Psychosocial intervention |
| --- | --- | --- | --- |
| No | No | 1.00 (Ref.) | 1.00 (Ref.) |
| No | Yes | 0.80 (0.42 to 1.54) | 0.69 (0.34 to 1.38) |
| Yes | No | 1.28 (0.94 to 1.73) | 0.71 (0.56 to 0.89) |
| Yes | Yes | 1.09 (0.74 to 1.60) | 0.57 (0.46 to 0.70) |

See footnote of **Figure 3** for further details

**OTHER GROUP MEMBERS (NON-AUTHOR CONTRIBUTORS) LIST**

“QUADIM project” working group (Italian Ministry of Health, Prevention Dept):

- *Italian Ministry of Health, General Directorate for Health Prevention*: Teresa DI FIANDRA, Natalia MAGLIOCCHETTI
- *Department of Mental Health, Lecco Hospital, Lecco, Italy*: Antonio LORA, Miriam BARRI
- *Emilia-Romagna Region*: Alessio SAPONARO
- *Lazio Region*: Andrea GADDINI, Valentina MATTIA
- *Sicily Region*: Salvatore SCONDOTTO, Walter POLLINA ADDARIO, Marco BERARDI, Monica DI GIORGI
- *University of Milano-Bicocca, Dept. of Statistics and Quantitative Methods*: Giovanni CORRAO, Matteo MONZIO COMPAGNONI
- *IRCCS Mario Negri*: Angelo BARBATO, Barbara D’AVANZO, Igor MONTI
- *SDA Cergas Bocconi*: Valeria TOZZI, Lucia FERRARA

“Monitoring and assessing care pathways (MAP)” working group (Italian Ministry of Health)

- *Polytechnic University of Marche (coordinator)*: Flavia CARLE, Andrea BUCCI, Chiara CASOLI, Marianxhela DAJKO.
- *Italian Ministry of Health*: *Dept of Health Planning*: Donata BELLENTANI, Simona CARBONE, Carla CECCOLINI, Angela DE FEO, Cristina GIORDANI, Lucia LISPI, Rosanna MARINIELLO, Federica MEDICI, Paola PISANTI, Modesta VISCA, Rinaldo ZANINI; *General Directorate for Health Prevention*: Teresa DI FIANDRA, Natalia MAGLIOCCHETTI, Giovanna ROMANO
- *University of Milano-Bicocca, Laboratory of Healthcare Research & Pharmacoepidemiology*: Anna CANTARUTTI, Giovanni CORRAO, Pietro PUGNI, Federico REA
- *Department of Epidemiology Lazio Region*: Marina Davoli, Mirko DI MARTINO,
- *Aosta Valley Region*: Patrizia VITTORI, Giuliana Vuillermin
- *Campania Region*: Alfonso Bernardo, Anna Fusciante
- *Emilia Romagna Region*: Laura BELOTTI, Rossana DE PALMA, Enza DI FELICE
- *Friuli Venezia Giulia Region*: Andrea DI LENARDA, Marisa PREZZA
- *Lazio Region*: Danilo FUSCO, Adele LALLO, Chiara MARINACCI
- *Lombardy Region*: Roberto BLACO, Olivia LEONI, Antonio LORA
- *Marche Region*: Liana SPAZZAFUMO, Simone PIZZI
- *Molise Region*: Maria SIMIELE, Giuseppe MASSARO
- *Puglia Region*: Ettore ATTOLINI, Vito LEPORE, Vito PETRAROLO
- *Sicily Region*: Salvatore SCONDOTTO, Giovanni DE LUCA
- *Tuscany Region*: Paolo FRANCESCONI, Carla RIZZUTO
- *Veneto Region*: Francesco AVOSSA, Silvia VIGNA
- *Research and Health Foundation (Fondazione Res -Ricerca e Salute-)*: Letizia DONDI, Nello MARTINI, Antonella PEDRINI, Carlo PICCINNI
- *National Agency for Regional Health Services*: Mimma COSENTINO, Maria Grazia MARVULLI
- *ANMCO (National Association of Hospital Cardiologists) Study Center*: Aldo MAGGIONI
