# Supplementary figures and images for: Does the mental health system provide effective coverage to people with schizophrenic disorder? A self-controlled case series study in Italy
Source: Soc Psychiatry Psychiatr Epidemiol. 2021 Jun 16;57(3):519–29. doi: 10.1007/s00127-021-02114-9 (PMC8934324; doi:10.1007/s00127-021-02114-9)

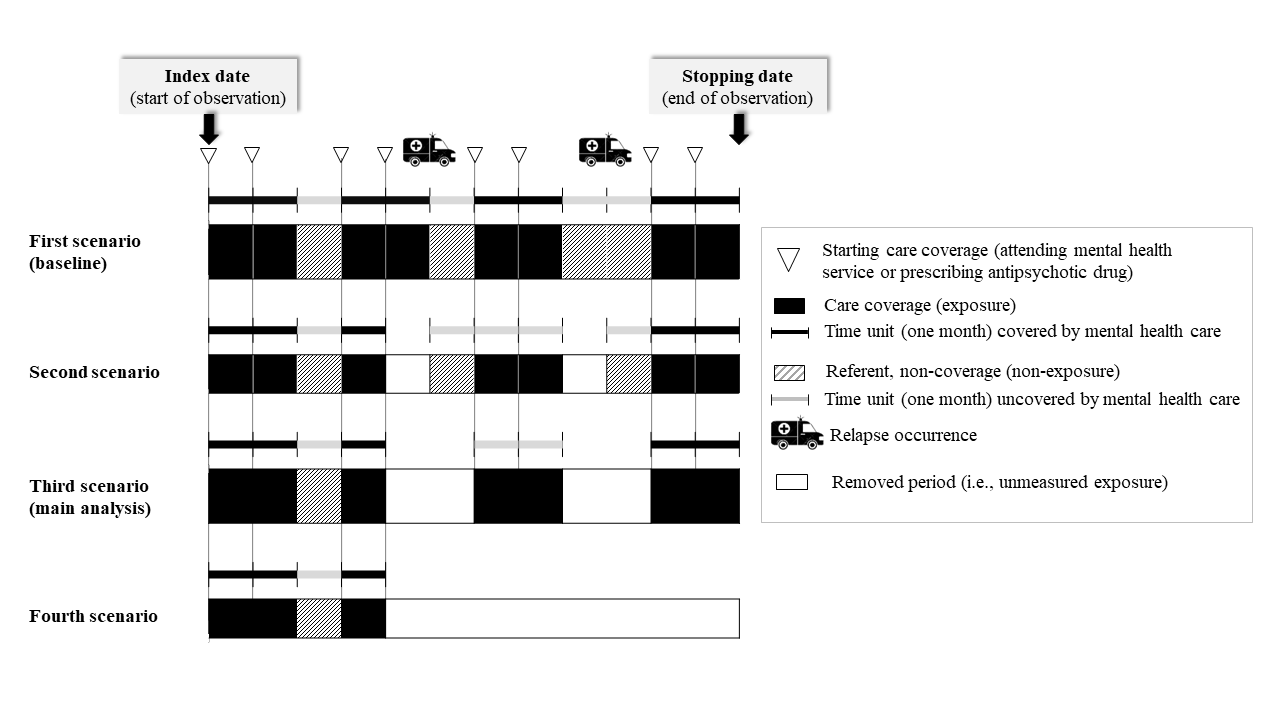

Supplement: Supplementary file 2 — Supplementary file2 (TIF 128 KB) [file 127_2021_2114_MOESM2_ESM.tif]

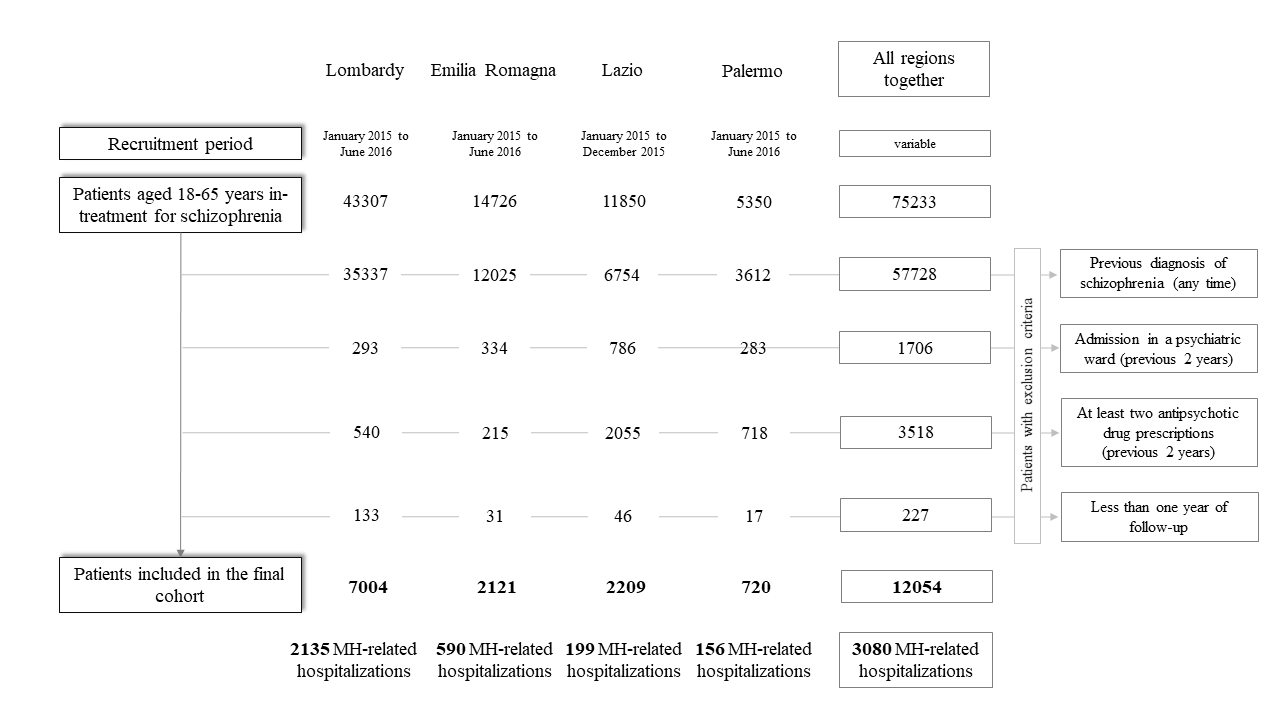

Supplement: Supplementary file 3 — Supplementary file3 (TIF 117 KB) [file 127_2021_2114_MOESM3_ESM.tif]
